# Flexible Nanocellulose/Lignosulfonates Ion-Conducting Separators for Polymer Electrolyte Fuel Cells

**DOI:** 10.3390/nano10091713

**Published:** 2020-08-29

**Authors:** Carla Vilela, João D. Morais, Ana Cristina Q. Silva, Daniel Muñoz-Gil, Filipe M. L. Figueiredo, Armando J. D. Silvestre, Carmen S. R. Freire

**Affiliations:** 1 Department of Chemistry, CICECO-Aveiro Institute of Materials, University of Aveiro, 3810-193 Aveiro, Portugal; jmorais@ua.pt (J.D.M.); ana.cristina.silva@ua.pt (A.C.Q.S.); armsil@ua.pt (A.J.D.S.); 2 Department of Materials and Ceramic Engineering, CICECO-Aveiro Institute of Materials, University of Aveiro, 3810-193 Aveiro, Portugal; danielmg@ua.pt (D.M.-G.); lebre@ua.pt (F.M.L.F.)

**Keywords:** bacterial nanocellulose, lignosulfonates, mechanical performance, thermal-oxidative stability, ion-exchange membranes, biobased separators, ionic conductivity

## Abstract

The utilization of biobased materials for the fabrication of naturally derived ion-exchange membranes is breezing a path to sustainable separators for polymer electrolyte fuel cells (PEFCs). In this investigation, bacterial nanocellulose (BNC, a bacterial polysaccharide) and lignosulfonates (LS, a by-product of the sulfite pulping process), were blended by diffusion of an aqueous solution of the lignin derivative and of the natural-based cross-linker tannic acid into the wet BNC nanofibrous three-dimensional structure, to produce fully biobased ion-exchange membranes. These freestanding separators exhibited good thermal-oxidative stability of up to about 200 °C, in both inert and oxidative atmospheres (N_2_ and O_2_, respectively), high mechanical properties with a maximum Young’s modulus of around 8.2 GPa, as well as good moisture-uptake capacity with a maximum value of ca. 78% after 48 h for the membrane with the higher LS content. Moreover, the combination of the conducting LS with the mechanically robust BNC conveyed ionic conductivity to the membranes, namely a maximum of 23 mS cm^−1^ at 94 °C and 98% relative humidity (RH) (in-plane configuration), that increased with increasing RH. Hence, these robust water-mediated ion conductors represent an environmentally friendly alternative to the conventional ion-exchange membranes for application in PEFCs.

## 1. Introduction

The increasing awareness toward clean energy and environmentally friendly materials is imposing a societal shift to meet the targets of the 2030 Agenda for Sustainable Development. Thus, the utilization of renewable raw materials for the development of the key components of fuel cells, which are efficient energy conversion technologies with zero-to-low emissions [1], is being explored to soften the impact of their production. Within the deluge of renewable raw materials, nanocellulose is one of the most interesting candidates to construct both the ion-exchange membrane and the electrodes for polymer electrolyte fuel cells (PEFCs), given its renewable nature, anisotropic shape, tailorable surface chemistry, and excellent mechanical properties, as recently reviewed by Vilela et al. [2]. In fact, bacterial nanocellulose (BNC), viz. the biotechnologically produced nanoscale form of cellulose [3,4], is particularly suitable, given its ability to be biosynthesized directly in the form of membranes with an adjustable size and shape, and also because of its unique mechanical performance. However, BNC presents a very low ionic conductivity [5,6], and therefore, the majority of the studies deal with either the chemical modification of BNC to introduce ionic moieties (e.g., sulfonic acid groups [7]) or the combination of BNC with synthetic polyelectrolytes (e.g., poly(bis[2-(methacryloyloxy)ethyl] phosphate) [8] and poly(4-styrene sulfonic acid) [9,10]) or ionomers (e.g., Nafion^®^ [11,12]) that enable the transport of ions [2].

The demand for fully biobased ion-exchange membranes has already prompted the fabrication of, for example, ion-exchange membranes composed of chondroitin sulfate (a sulfated glycosaminoglycan) [13], cellulose nanocrystals obtained by acidic hydrolysis with sulfuric acid (a sulfated nanocellulose) [14], and fucoidan (a sulfated polysaccharide) combined with BNC [6]. In all these instances, the adsorption of water molecules assisted by the sulfate moieties produced paths for the structural diffusion of protons, which translated into separators with ionic conductivity. Lignosulfonates (LS), which are water-soluble anionic sulfonated lignin derivatives obtained as by-products of the sulfite pulping process [15,16], present a high content of sulfonate groups (sulfur content: 3.5–8.0 wt.% [17]) and, therefore, are strong contenders for non-expensive biobased ion conducting materials. Nevertheless, the high-water solubility of LS, and their non-film forming ability are major constraints for application in a PEFC that generates water and heat as reaction by-products. Although LS has already been blended with, for instance, poly(benzimidazole) [18], poly(sulfone) [19] and poly(styrene sulfonate)/nano-silica [20] for application in fuel cells, and BNC was previously biosynthesized in the presence of LS to assess the effect of the lignin derivative on the physical properties of BNC [21], the combination between LS and BNC has not yet been explored to fabricate biobased ion-exchange separators for PEFCs.

In this manner, the present study envisages the assembly and characterization of biobased separators composed of BNC and LS, for potential application as ion-exchange membranes. These naturally derived separators were assembled via diffusion of an aqueous solution of LS and tannic acid (TA, acting as a natural cross-linker), into the wet BNC nanofibrous three-dimensional structure. The resultant membrane separators were characterized in terms of structure (infrared spectroscopy), composition (energy dispersive X-ray spectrometry), morphology (scanning electron microscopy), thermal-oxidative stability (thermogravimetric analysis), mechanical performance (tensile tests), moisture-uptake capacity, ionic conductivity (impedance spectroscopy), and always compared with ion-exchange membranes reported in literature.

## 2. Materials and Methods

### 2.1. Chemicals and Materials

Lignosulfonic acid sodium salt (LS, Mw ~52,000 and Mn ~7000) and tannic acid (TA, C_76_H_52_O_46_, from Chinese natural gall nuts) were acquired from Sigma-Aldrich (St. Louis, MO, USA). Ultrapure water (Type 1, 18.2 MΩ cm at 25 °C) was purified by a Simplicity^®^ Water Purification System (Merck, Darmstadt, Germany). Additional chemicals or solvents were of laboratory grade.

Bacterial nanocellulose (BNC), entailing a three-dimensional network of nano- and micro-fibrils with 10–200 nm width, was biosynthesized in the form of wet membranes (99.5% of water) by the *Gluconacetobacter sacchari* bacterial strain [6].

### 2.2. Preparation of the BNC/LS-Based Membranes

BNC membranes in the wet state with a diameter of about 70 mm and 40% water content were placed on a Petri-dish having an aqueous solution of LS (2:1 and 4:3 mass fraction of BNC:LS, selected based on a previous study [6]) and TA (20% *w*/*w* relative to LS, chosen based on a previous study [6]), as summarized in Table 1. Following the complete absorption of the solutions (viz. 100% entrapment efficiency) at room temperature, the membranes were placed in a ventilated oven (Thermo Fisher Scientific, Waltham, MA, USA) at 105 °C for 24 h to facilitate the thermal cross-linking during the drying process. All membrane separators were produced in triplicates and stored in desiccators.

### 2.3. Characterization Methods

#### 2.3.1. Thickness

A hand-held coolant proof digimatic micrometer MDC-25PX (Mitutoyo Corporation, Tokyo, Japan) was utilized to quantify the thickness at ten random sites of the membrane separators.

#### 2.3.2. Attenuated Total Reflection-Fourier Transform Infrared (ATR-FTIR) Spectroscopy

A Perkin-Elmer FT-IR System Spectrum BX spectrophotometer (Perkin-Elmer Inc., Waltham, MA, USA) fitted out with a single horizontal Golden Gate ATR cell (Specac^®^, London, UK) was used to compute the ATR-FTIR spectra in the range of 600–4000 cm^−1^ at a resolution of 4 cm^−1^ over 32 scans.

#### 2.3.3. Scanning Electron Microscopy (SEM) Combined with Energy Dispersive X-ray Spectroscopy (EDS)

An ultra-high-resolution field-emission HR-FESEM Hitachi SU-70 microscope (Hitachi High-Technologies Corporation, Tokyo, Japan), equipped with a microanalysis Bruker QUANTAX 400 detector for EDS (Bruker Nano GmbH, Berlin, Germany), was utilized to acquire micrographs of the membranes and evaluate their elemental chemical composition. Prior to analysis, the test specimens for surface and cross-section (fractured in liquid nitrogen) examination were put on a steel plate and coated with a carbon film.

#### 2.3.4. Thermogravimetric Analysis (TGA)

A SETSYS Setaram TGA analyzer (SETARAM Instrumentation, Lyon, France) equipped with a platinum cell was used to assess the thermal stability. The test specimens were heated from 25 to 800 °C with a heating rate of 10 °C min^−1^ under two distinct atmospheres, namely nitrogen and oxygen.

#### 2.3.5. Tensile Testing

A uniaxial Instron 5566 testing machine (Instron Corporation, Norwood, MA, USA) was utilized for the tensile tests in the traction mode at a crosshead velocity of 10 mm min^−1^ using a 500 N static load cell. The rectangular test specimens (50 × 10 mm^2^) were formerly dried at 40 °C and all measurements were conducted on five replicates.

#### 2.3.6. Moisture-Uptake Capacity

The moisture-uptake was quantified by putting the dry test specimens (20 × 20 mm^2^) in a conditioned cabinet with 98% relative humidity (RH) (saturated potassium sulphate aqueous solution, 97.6 ± 0.5% [22]) at room temperature for 48 h. After taking the test specimens from the cabinet, the weight (*W_w_*) was measured and the moisture-uptake capacity was determined as:

$$\mathrm{Moisture}-\mathrm{uptake}\;\left(\%\right)=\left(W_{w}-W_{0}\right)\times W_{0}^{-1}\times 100$$

where *W*_0_ is the initial weight of the dry membrane.

#### 2.3.7. Ionic Conductivity

Impedance spectroscopy (Agilent (County of Santa Clara, CA, USA) E4980A Precision LCR meter) was utilized to quantify the in-plane (IP) ionic conductivity (σ) at different temperature (40 °C to 94 °C) and RH (30% to 98%) conditions in an ACS Discovery DY110 climatic chamber (Angelantoni Test Technologies Srl, Massa Martana, Italy). The measurements were conducted on rectangular test specimens (ca. 15 × 5 mm^2^) whereon two stripes of silver paste (Agar Scientific, Essex, UK) distancing ca. 10 mm were painted. Moreover, a pseudo 4-electrode configuration in a tubular sample holder was applied to guarantee full exposure of the test specimen surface to the controlled atmosphere and to give the necessary electrical contact between the test specimen and the LCR meter. The impedance spectra were collected between 20 Hz and 2 × 10^6^ Hz with a test signal amplitude of 100 mV and analyzed with the ZView software (Version 2.6b, Scribner Associates (Southern Pines, NC, USA)) to calculate the Ohmic resistance (R) of the test specimen. The conductivity was then determined by the equation: $\sigma =L_{0}{\left(R\delta \omega \right)}^{-1}$, where *L*_0_ is the distance between the two silver stripes, *δ* is the thickness of the membrane, and *w* is the width of the membrane.

## 3. Results and Discussion

### 3.1. Membrane Production and Characterization (Structure and Morphology)

**Figure 1:**
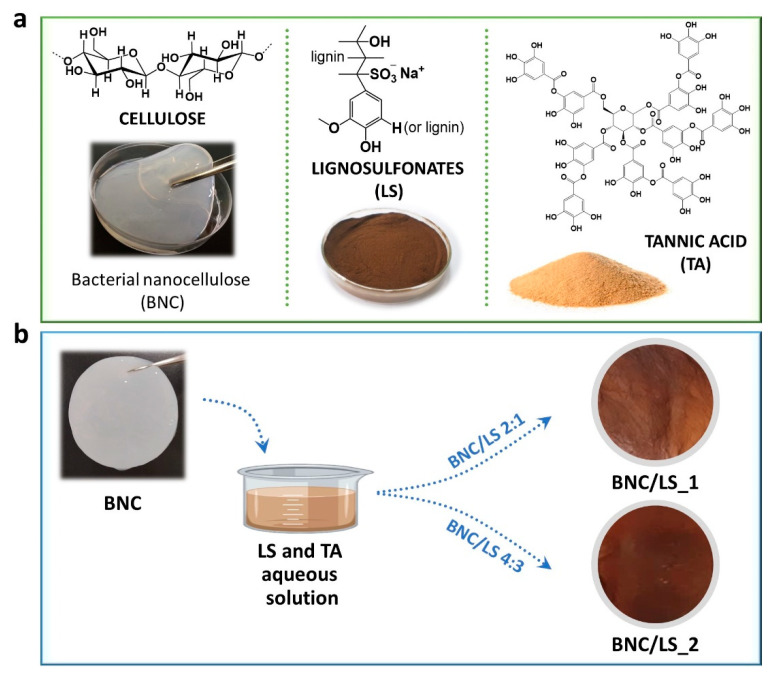
(**a**) Chemical structure, and photographs of the precursors, namely bacterial nanocellulose (BNC, wet membrane), lignosulfonates (LS, powder) and tannic acid (TA, powder), and (**b**) route for the production of the BNC/LS-based membranes and the photographs of the respective dry membranes.

Biobased ion-exchange separators were fabricated by combining a nanocellulose substrate, namely BNC, with a phenolic natural-based polyelectrolyte, namely LS, as outlined in Figure 1a. The straightforward diffusion of an aqueous solution of LS and TA (natural-based cross-linker) into the never-dried BNC nanofibrous three-dimensional structure, produced brownish BNC/LS-based membranes (Figure 1b), because of the signature dark brown color of the LS polyelectrolyte. BNC was picked for its in situ-moldability as a three-dimensional porous membrane, as well as good thermal stability and mechanical performance [23], while the lignin derivative (LS) was chosen for its high content of sulfonate groups ($-SO_{3}^{-}$) that facilitate ion motion and, hence, exhibits ionic conductivity [19]. On the other hand, the natural phenolic TA was selected for its cross-linking capability toward LS [24], as well as other neutral or charged macromolecules via physical or chemical interactions [25,26,27], to enable the retention of the water-soluble LS inside the wet BNC nanofibrous porous structure, as reported for other BNC-based membranes [6]. The resultant BNC/LS-based separators have two different compositions, namely 395 ± 16 mg of LS per cm^3^ of membrane for the BNC/LS_1, and 547 ± 19 mg cm^−3^ for the BNC/LS_2, and thus, the thickness of the membranes increased with the increasing content of LS, as observed in Table 1.

**Figure 2:**
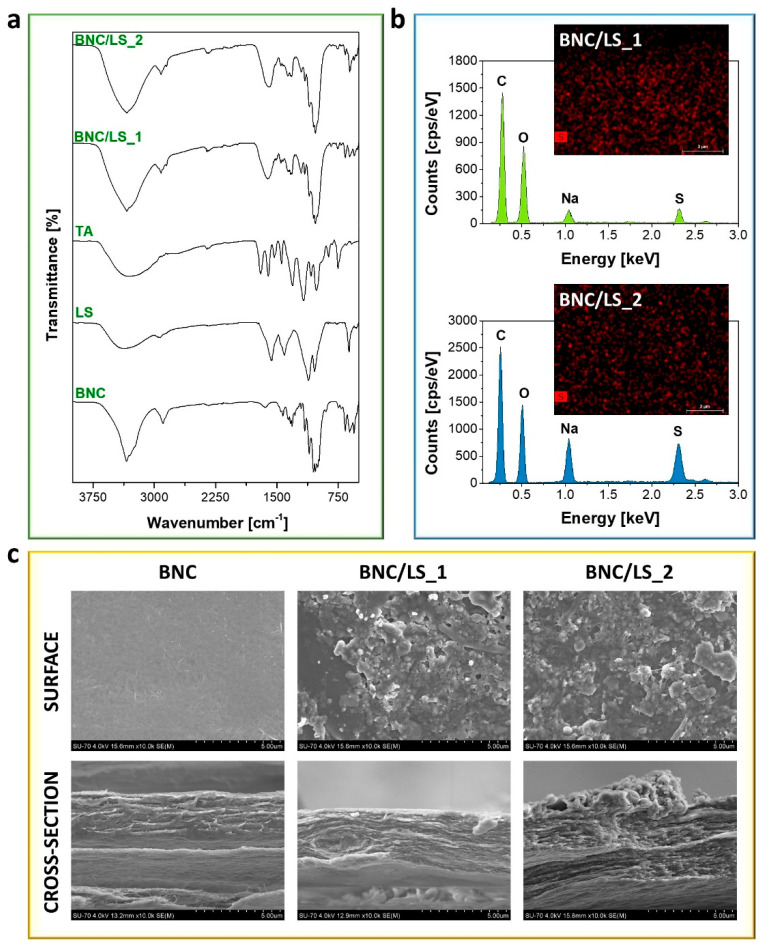
(**a**) Attenuated total reflection-Fourier transform infrared (ATR-FTIR) spectra of BNC, LS, TA, and BNC/LS-based membranes, (**b**) EDS spectra and mapping (scale bar: 3 μm) of the cross-section of the two BNC/LS membranes, and (**c**) SEM micrographs of the surface and cross-section of the pristine BNC and BNC/LS-based membranes (×10.0 k magnification).

The structural characterization of the membrane separators was carried out by ATR-FTIR vibrational spectroscopy. According to Figure 2a, the spectrum of the pristine bacterial polysaccharide displays the cellulose characteristic absorption bands at about 3341 cm^−1^ (O–H stretching), 2893 cm^−1^ (C–H stretching), 1314 cm^−1^ (O–H in plane bending), 1160 cm^−1^ (C–O–C antisymmetric stretching), and 1031 cm^−1^ (C–O stretching) [28,29]. The ATR-FTIR spectrum of LS (Figure 2a) exhibits the usual structural pattern of this lignin derivative with the presence of the absorption bands at around 3368 cm^−1^ (O–H stretching), 1570 cm^−1^ (C=C aromatic skeletal vibrations), 1410 cm^−1^ (C–O stretching), 1114, and 1040 cm^−1^ (S=O asymmetric and symmetric stretching), and 618 cm^−1^ (C–S stretching) [30,31]. The ATR-FTIR spectrum of TA (Figure 2a) presents the common absorption bands of an aromatic phenolic compound at about 3306 cm^−1^ (O–H stretching), 1700 cm^−1^ (C=O stretching), 1606 cm^−1^ (C–C aromatic stretching), 1308 cm^−1^ (C_ar_–OC stretching, C_ar_–O–H in-plane bending, C–C aromatic stretching), and 1174 cm^−1^ (O–CO and C_ar_–CO stretching, C_ar_–O–H in-plane bending) [32].

Additionally, the FTIR-ATR spectra of both BNC/LS-based membranes (Figure 2a) show similarities with those of their precursors, particularly with BNC and LS. Although most of the absorption bands of LS and TA are entirely overlapped with the vibrations of the dominant component, viz. BNC, the efficient inclusion of LS into the BNC nanofibrous three-dimensional structure was clearly confirmed.

The elemental chemical composition of the BNC/LS-based membranes was assessed by EDS analysis, as depicted in Figure 2b. The EDS spectra of the two biobased separators confirm the existence of BNC and LS through the detection of the sulfur (S), sodium (Na), oxygen (O), and carbon (C) peaks at 2.31, 1.04, 0.51, and 0.27 keV, respectively. Predictably, the presence of sodium shows the bound of the sulfonate moieties to the Na cations. The analysis of both EDS spectra demonstrates that the sulfur content of the BNC/LS_1 membrane is lower than that of BNC/LS_2, which is in line with the relative contents of BNC and LS used in their preparation. Furthermore, the EDS mapping of sulfur at the surface of both membrane separators showed an appreciable content and a uniform distribution of the element (Figure 2b, inset images with sulfur element in red color). Therefore, the lignin derivative was effectively incorporated into the BNC porous three-dimensional network, as already verified by infrared spectroscopy.

The morphology of the membrane separators was examined by SEM with the surface and cross-section micrographs of BNC and BNC/LS-based membranes shown in Figure 2c. A glimpse over the micrographs of the surface and cross-section of the pristine BNC membrane evidences the morphological traits of this nanocellulose substrate, namely the well-known nanofibrillar and lamellar microstructure [23]. In Figure 2c, it is further noticeable that the inclusion of the sulfonated lignin derivative camouflaged the nanofibrils and occupied the lamellar spaces of the BNC porous network, which is particularly notorious for the membrane containing the higher LS content (547 ± 19 mg cm^−3^, BNC/LS_2, Table 1). This behavior is quite common as in fact documented for other partially and fully biobased BNC-based ion-exchange separators [5,6,8].

### 3.2. Thermal and Mechanical Properties

**Figure 3:**
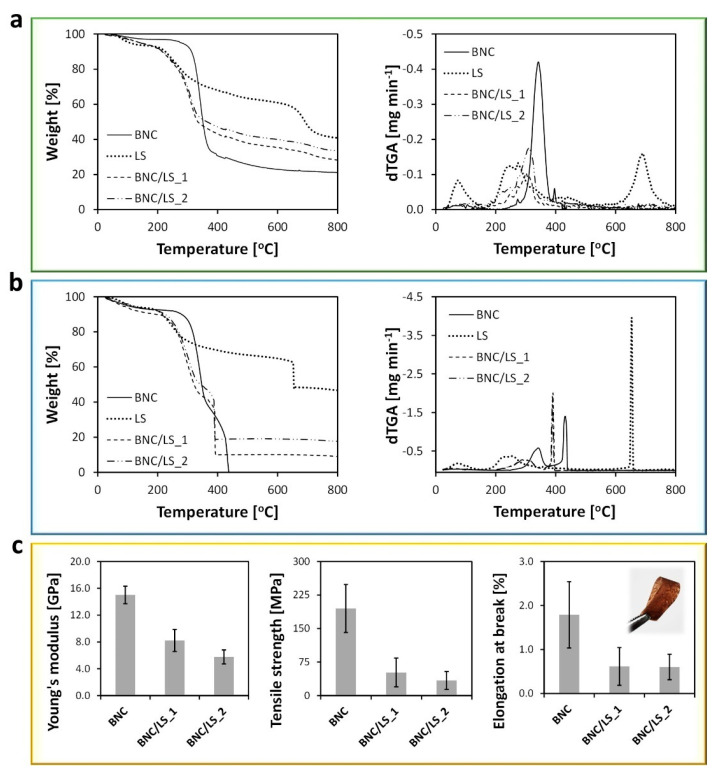
(**a**,**b**) Thermograms (left) with the respective derivatives (right) of pristine BNC, LS, and BNC/LS-based membranes under (**a**) N_2_ (inert) and (**b**) O_2_ (oxidative) atmospheres, and (**c**) tensile tests data: Young’s modulus (left), tensile strength (middle), and elongation at break (right, the inset photograph corresponds to BNC/LS_1) of the pristine BNC and BNC/LS-based membranes.

Thermogravimetric analysis (TGA) was utilized to investigate the thermal-oxidative stability of the BNC/LS-based membranes, along with the pristine BNC and LS samples. The degradation profile of BNC (Figure 3a) under inert atmosphere follows a single weight-loss stage with maximum decomposition temperature of 350 °C, as a consequence of the pyrolysis of the cellulose skeleton [33]. On the other hand, the LS exhibits a degradation profile composed of two weight-loss stages (Figure 3a), while the initial water evaporation below 100 °C. The first weight-loss step occurs at a maximum decomposition temperature of 270 °C, allocated to the pyrolysis of oxygen-containing groups, while the second stage appears at a maximum decomposition temperature of 695 °C and is ascribed to the loss of the remaining O_2_-containing groups on carbon edges [34].

The thermograms of the two BNC/LS-based membranes under N_2_ atmosphere (Figure 3a) present a similar profile with one weight-loss step with maximum rate of decomposition temperatures at 314 °C for BNC/LS_1 and 318 °C for BNC/LS_2, and a residue at 800 °C that increased with the LS content from 28% for BNC/LS_1 to 32% for BNC/LS_2. So, the combination between LS and BNC created membrane separators with lower thermal stability when compared with the pristine BNC, because of the presence of the less thermally stable, and amorphous LS polyelectrolyte (Figure 3a). Similar results were obtained for membranes composed of BNC and fucoidan, where the inclusion of the sulphated polysaccharide into the BNC network also yielded materials with lower thermal stability than the pristine BNC [6].

When applied in a PEFC, the BNC/LS-based membranes will have to withstand an oxidative environment, therefore, their thermal-oxidative stability was also measured under oxidative atmosphere (Figure 3b). For the pristine BNC membrane, the process in oxygen is marked by a two-stage degradation profile with maximum rate of decomposition temperatures at ca. 340 and 433 °C, reaching a complete degradation with no residue [35,36]. For the LS powder, the thermogram is composed by two weight-loss stages with maximum rate of decomposition temperatures at around 246 and 653 °C, while the loss of water below 100 °C (loss of ca. 5%), leaving a residue of about 46% at 800 °C. This profile is roughly equivalent to the process under N_2_ atmosphere, both in terms of temperatures and the total residue content at 800 °C.

The TGA tracing of the BNC/LS-based membranes (Figure 3b), contrary to the process in N_2_, displays two weight-loss stages with maximum rate of decomposition temperatures at around 310 and 391 °C for BNC/LS_1, and 318 and 389 °C for BNC/LS_2, with residues of 9 and 18% of the initial mass at 800 °C for BNC/LS_1 and BNC/LS_2, respectively. Although the BNC/LS-based membranes present a lower thermal stability than the commercial Nafion^®^ ionomer used in PEFCs (ca. 290 °C [12]), both membranes are thermally stable at least up to 200 °C in both inert and oxidative atmospheres. Hence, their thermal-oxidative profile does not jeopardize the envisioned application as ion-exchange membranes for PEFCs that operate under temperatures below 100 °C.

The mechanical performance of the BNC/LS-based membranes was investigated by tensile tests and the respective data are compiled in Figure 3c. The membrane composed solely of BNC (thickness: 69 ± 11 μm) presents values of Young’s modulus of 15.0 ± 1.3 GPa, tensile strength of 195 ± 54 MPa and elongation at break of 1.8 ± 0.8%, which are in tune with data reported elsewhere [36]. No values were obtained for the LS since this amorphous polymer is not a film-forming material. On the other hand, the incorporation of LS into the nanostructured BNC, produced membrane separators with lower mechanical performance when compared with the pristine BNC, but with the advantage that the pure LS does not form free-standing films. The BNC/LS_1 membrane exhibits values of Young’s modulus of 8.2 ± 1.6 GPa, tensile strength of 52 ± 32 MPa, and elongation at break of 0.6 ± 0.4%, whereas the BNC/LS_2 membrane presents a Young’s modulus of 5.8 ± 1.1 GPa, tensile strength of 34 ± 20 MPa and elongation at break of 0.6 ± 0.3% (Figure 3c). Although the reduction of these three mechanical parameters translates into less stiffer materials, the membranes are bendable (see the inset photograph in Figure 3c) and still adequate for application as ion separators for PEFCs.

When compared with the ion-exchange membranes reported in literature, the BNC/LS-based membrane separators developed in the present study possess lower mechanical performance than, e.g., the membranes constituted by BNC combined with poly(4-styrene sulfonic acid), which is a synthetic polyelectrolyte containing sulfonic acid moieties [10]. Nevertheless, their mechanical performance is comparable, for example, with those of membranes composed of poly(benzimidazole) and LS [18], and poly(styrene sulfonate), LS and nano-silica [20], but most importantly they present superior mechanical properties than the reference benchmark Nafion^®^ membrane with a Young’s modulus of 0.25 GPa and tensile strength of 43 MPa [37].

### 3.3. Moisture-Uptake Capacity and Ionic Conductivity

**Figure 4:**
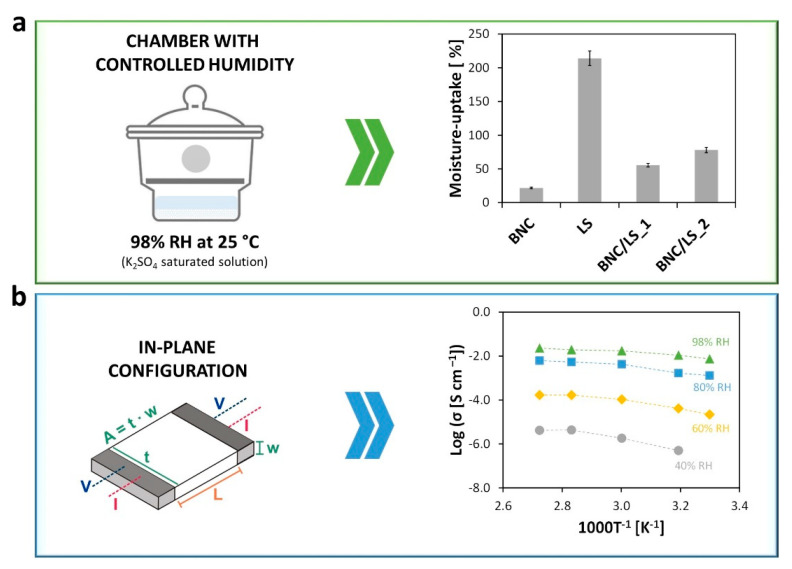
(**a**) Moisture-uptake capacity of the pristine BNC, LS, and BNC/LS-based membranes after 48 h at room temperature and 98% relative humidity (RH) in a chamber with controlled humidity, and (**b**) Arrhenius-type plot of the ionic conductivity (σ) of BNC/LS_2 membrane at different RH (40%, 60%, 80%, and 98%) in the in-plane configuration [5].

The moisture-uptake capacity of the BNC/LS-based membranes, as well as of the pristine BNC membrane and LS powder, was estimated by positioning the materials in a chamber with controlled humidity, namely 98% relative humidity (RH), during 48 h. Predictably, both BNC and LS have the aptitude to absorb environmental humidity, although with distinct values, i.e., 21.8 ± 2.1% for BNC [6] and 214.0 ± 5.7% for LS, given their different natures. The two BNC/LS-based membranes absorbed moisture with values of 55.6 ± 2.4% for BNC/LS_1 and 78.0 ± 3.7% for BNC/LS_2, as epitomized in Figure 4a. Furthermore, both membranes guarded their mechanical integrity after the moisture absorption tests, confirming the good wet-dimensional stability of BNC. The values obtained for both separators are superior than the water absorption of the benchmark Nafion^®^ (~38%, when fully hydrated at 100 °C for 1 h) [37]. So, the inclusion of LS is clearly beneficial toward the augment of the moisture-uptake capacity that actively affects the ionic conductivity [38]. Understandably, the data show that the adsorption of water molecules is supported by the sulfonate groups, which will produce, without a doubt, paths for the structural diffusion of ions [2].

In this perspective, the ionic conductivity (σ) of the Na-form membrane with the higher LS content (i.e., BNC/LS_2) was measured by impedance spectroscopy in the in-plane configuration (Figure 4b). Previous studies have shown that the pristine BNC is a poor ionic conductor (63 μS cm^−1^ at 98% RH and 94 °C) [5,6], just like the other nanofibrillar form of cellulose, viz., cellulose nanofibrils (50 μS cm^−1^ at nominal 100% RH and 100 °C) [14]. Nevertheless, when combined with a lignin derivative with a polyelectrolytic nature, namely LS, the BNC membranes turns into an ion-conducting material. In fact, the BNC/LS_2 membrane exhibits ionic conductivity that increases with the rise in relative humidity (40–98% RH) and temperature (30–94 °C), as depicted in the Arrhenius-type plot of Figure 4b. Inevitably, the RH is the factor that primarily affects the ionic conductivity in the case of water-mediated ionic conductors [2].

According to Table 2, the ionic conductivity increases up to five orders of magnitude when the RH rises from 40% to 98%, and only one order of magnitude when the temperature varies from 30 to 94 °C. For instance, the conductivity increased from 5.0 × 10^−7^ S cm^−1^ at 40% RH and 40 °C to 1.1 × 10^−2^ S cm^−1^ at 98% RH and 40 °C, while it only increased from 2.2 × 10^−5^ S cm^−1^ at 30 °C and 60% RH to 1.7 × 10^−4^ S cm^−1^ at 40 °C and 60% RH. Furthermore, the maximum ionic conductivity was reached at elevated humidity and temperature conditions (98% RH and 94 °C) with a value of 23 mS cm^−1^. After the measurements at 98% RH and 94 °C, no apparent degradation is visible, and the membrane withheld its mechanical integrity.

Herein, it should be noted that for lower RH the ionic conductivity is focused essentially on Na^+^ ion transport, while at higher RH there is probably some exchange of Na^+^ ions by protons, and the ionic conductivity might be a binary cation system focused on proton and Na^+^ ion conduction. In this context, Saito et al. [39] studied the mechanisms of ion and water transport of the commercial Nafion^®^ membranes in the H- and Na-forms. According to the authors, both forms presented ionic conductivity that improved with increasing water content of the membranes, and the ionic conductivity of the H-form membrane (~150 mS cm^−1^, fully hydrate state, 25 °C) is far greater than that of the Na-form membrane (~35 mS cm^−1^, fully hydrate state, 25 °C).

In addition, the dependency of the ionic conductivity on temperature shows a slight bending that indicates a reduction of the apparent activation energy with the increase in temperature (Figure 4b). Such type of Vogel–Tammann–Fulcher (VTF) behavior indicates a role of the segmental motion in assisting ion transport particularly under low humidity when sodium transport is expected to be relatively more important. At high humidity (80% RH and more), the proton concentration is the highest and the conductivity is expected to be essentially due to proton transport in the aqueous domains formed in the structure. In these conditions, the dependency of the ionic conductivity on temperature can be described by the Arrhenius relationship: $\sigma =\sigma_{0}e^{-E_{a}{\left(RT\right)}^{-1}}$ ($\sigma_{0}$—pre-exponential term, $E_{a}$—activation energy, *R*—gas constant, *T*—absolute temperature [9,40]), with an estimated $E_{a\;}$ for ion transport of ca. 26 kJ mol^−1^ at 80% RH and 18 kJ mol^−1^ at 98% RH. These values paralleled with those typically recorded for BNC [6] and Nafion^®^ [41,42], suggesting they are governed by similar ion conduction mechanism(s) [43,44].

Albeit the lower ionic conductivity of BNC/LS_2 when compared with the standard Nafion^®^ [42,45], the BNC/LS_2 membrane developed in the present study displays conductivity values that are comparable and, in some cases, even higher than other partially and fully biobased ion-exchange membranes reported in literature, as outlined in Table 3. Those examples include the membranes composed of CNCs, chitosan, and poly(vinyl alcohol) with a highest conductivity of 0.642 mS cm^−1^ (25 °C, fully hydrated) [46], the BNC/fucoidan membrane with a highest conductivity of 1.6 mS cm^−1^ (94 °C, 98% RH) [6], and the pure cellulose nanocrystals (CNCs) membrane with a highest conductivity of 2.5 mS cm^−1^ (90 °C, nominal 100% RH) [14]. On the other hand, it should be pointed out that there is a partially biobased membrane with an ionic conductivity that can reach up to 406 mS cm^−1^ (25 °C, fully hydrated), but only for the reason that three materials with high ionic conductivity, namely lignosulfonates, poly(styrene sulfonate), and nano-silica, are combined [20]. Nevertheless, these membranes composed of BNC and LS, with moderate ionic conductivity under variable temperature and humidity conditions, good mechanical performance, thermal-oxidative stability under inert and oxidative environments, and dimensional stability under humid conditions, show potential as an eco-friendly alternative of ion conductors for application in PEFCs.

## 4. Conclusions

The present study underlines the combination of two naturally derived polymeric materials, namely nanocellulose and a lignin derivative (lignosulfonates), to build fully biobased and ease-to-prepare ion-exchange membrane separators for utilization in polymer electrolyte fuel cells. The obtained freestanding membranes manifested appropriate thermal-oxidative stability up to about 200 °C in either N_2_ (inert) or O_2_ (oxidative) atmospheres, elevated mechanical properties with a maximum Young’s modulus of 8.2 GPa, along with good moisture-uptake capacity with a maximum value of ca. 78% after 48 h. Additionally, the blend of the conducting lignosulfonates with the mechanically robust bacterial nanocellulose granted a maximum ionic conductivity of 23 mS cm^−1^ at 94 °C and 98% RH to the membrane with the highest LS content. Therefore, these BNC/LS water-mediated ion conductors, with good mechanical performance, thermal-oxidative stability, and water-uptake capacity and whose conductivity is actively linked to humidification, can be employed as eco-friendly substitutes to ion-exchange membranes for application in PEFCs.

## Figures and Tables

**Table 1:** List of the prepared membranes with the corresponding composition and thickness values.

Membrane	*W*_BNC_:*W*_LS_^a^	*W*_LS_/*V*_total_ [mg cm^−3^] ^a^	Thickness [μm]
BNC	–	–	69 ± 11
BNC/LS_1	2:1	395 ± 16	75 ± 10
BNC/LS_2	4:3	547 ± 19	85 ± 9

^a^ composition was estimated by considering the dry weight of BNC (*W*_BNC_) and lignosulfonates (*W*_LS_), and the volume of the membrane (*V*_total_, determined by taking into account the diameter and thickness of the membranes); the values are expressed as mean ± standard deviation.

**Table 2:** Ionic conductivity values obtained for the BNC/LS_2 membrane at different temperature (30–94 °C) and relative humidity (RH, 40–98%).

Temperature [°C]	Ionic Conductivity [S cm^−1^]			
	40% RH	60% RH	80% RH	98% RH
30	–	2.2 × 10^−5^	1.3 × 10^−3^	7.3 × 10^−3^
40	5.0 × 10^−7^	4.1 × 10^−5^	1.7 × 10^−3^	1.1 × 10^−2^
60	1.8 × 10^−6^	1.1 × 10^−4^	4.2 × 10^−3^	1.7 × 10^−2^
80	4.3 × 10^−6^	1.7 × 10^−4^	5.4 × 10^−3^	1.9 × 10^−2^
94	4.1 × 10^−6^	1.7 × 10^−4^	6.1 × 10^−3^	2.3 × 10^−2^

**Table 3:** Examples of partially and fully biobased ion-exchange membranes reported in literature and compared with the present study.

	Components ^a^	Conductivity ^a^	Ref.
Partially biobased			
	CNCs/CH/PVA	0.642 mS cm^−1^ (IP, 25 °C, fully hydrated)	[46]
	CNFs/PBI	66.6 mS cm^−1^ (IP, 140 °C)	[47]
	BNC/Nafion^®^	140 mS cm^−1^ (IP, 94 °C, 98% RH)	[12]
	BNC/PSSA	185 mS cm^−1^ (IP, 94 °C, 98% RH)	[5]
	BNC/PMOEP	100 mS cm^−1^ (TP, 80 °C, 98% RH)	[35]
	BNC/P(bisMEP)	30 mS cm^−1^ (IP, 80 °C, 98% RH)	[8]
	k-carrageenan/IL	186 mS cm^−1^ (IP, 60 °C, 98% RH)	[48]
	LS/PBI	187 mS cm^−1^ (IP, 160 °C, anhydrous)	[18]
	LS/PSSA/nano-silica	406 mS cm^−1^ (TP, 25 °C, fully hydrated)	[20]
Fully biobased			
	BNC/LS	23 mS cm^−1^ (IP, 94 °C, 98% RH)	Present study
	BNC/Fucoidan	1.6 mS cm^−1^ (TP, 94 °C, 98% RH)	[6]
	CNCs	2.5 mS cm^−1^ (TP, 90 °C, 100% RH) 4.6 mS cm^−1^ (TP, 120 °C, 100% RH)	[14]
	Chondroitin sulfate/citric acid	37 mS cm^−1^ (TP, 25 °C, 98% RH)	[13]

^a^ BNC: bacterial nanocellulose, CH: chitosan, CNCs: cellulose nanocrystals, IL: ionic liquid (1-butyl-3-methyl-1H-imidazolium chloride ([Bmim]Cl)), IP: in-plane, LS: lignosulfonate, P(bisMEP): poly(bis[2-(methacryloyloxy)ethyl] phosphate), PBI: poly(benzimidazole), PMOEP: poly(methacryloyloxyethyl phosphate), PSSA: poly(4-styrene sulfonic acid), PVA: poly(vinyl alcohol), RH: relative humidity, TP: through-plane.
